# Supplementary material for: Modeling exercise using optogenetically contractible Drosophila larvae
Source: BMC Genomics. 2022 Aug 30;23:623. doi: 10.1186/s12864-022-08845-6 (PMC9425970; doi:10.1186/s12864-022-08845-6)
Supplement: Supplementary file 1 — Additional file 1: Fig. S1. The OMC setup and Scaled diagrams for the adapter ring setup. (A) Photograph showing the main parts of the OMC setup, which include the incubator, Arduino board, fly vial adapter, and the high-power LEDs within the vial adapter. Refer to Fig. 1A for more details. (B-B′) The two rings were stacked such that the cogs of the Fitted piece (B) overlap the troughs in the Base spacer (B′) and then joined using acrylic cement to generate the holder. The inner diameter of the rings is 0.97 in. (for B) and 0.90 in. (for B′). Note: The stroke width of the rings has been increased from 0.001 to 0.25 for a better visibility in the manuscript figure. For laser cutting, the lines were kept at a dimension of 0.001 in. Fig. S2. Establishment of an endurance training regimen using OMCs. A Representative larval body wall muscle showing the muscle segment A3, which is used for imaging. Body wall muscle is stained with Phalloidin 633 (far red). B w1118 larval body wall muscles stained with anti-glycogen antibody (α-Glycogen in grey), phalloidin-594 (red), and DAPI (blue). Sample in the lower panel was treated with amyloglucosidase for 20 min before staining with α-Glycogen antibody. C A schematic showing the experimental set up and frequency of OMCs used for modeling of endurance exercise. D Anti-Glycogen (α-Glycogen) staining of larval body wall muscles from control animals and animals that were endurance trained. Endurance trained animals show much stronger staining indicating increased glycogen storage in endurance trained muscles. E Anti-glycogen (α-Glycogen) staining of control and endurance trained larval body wall muscles. These are biological replicates (n = 8) pertaining to (D). Note that the images in the second column are represented in (D). Fig. S3. Acute exercise affects genes involved in tracheal development, wound healing, oxidative stress, and glutathione metabolism. A Principal Component Analysis (PCA) showing the separation of control and ex [file 12864_2022_8845_MOESM1_ESM.docx]

**
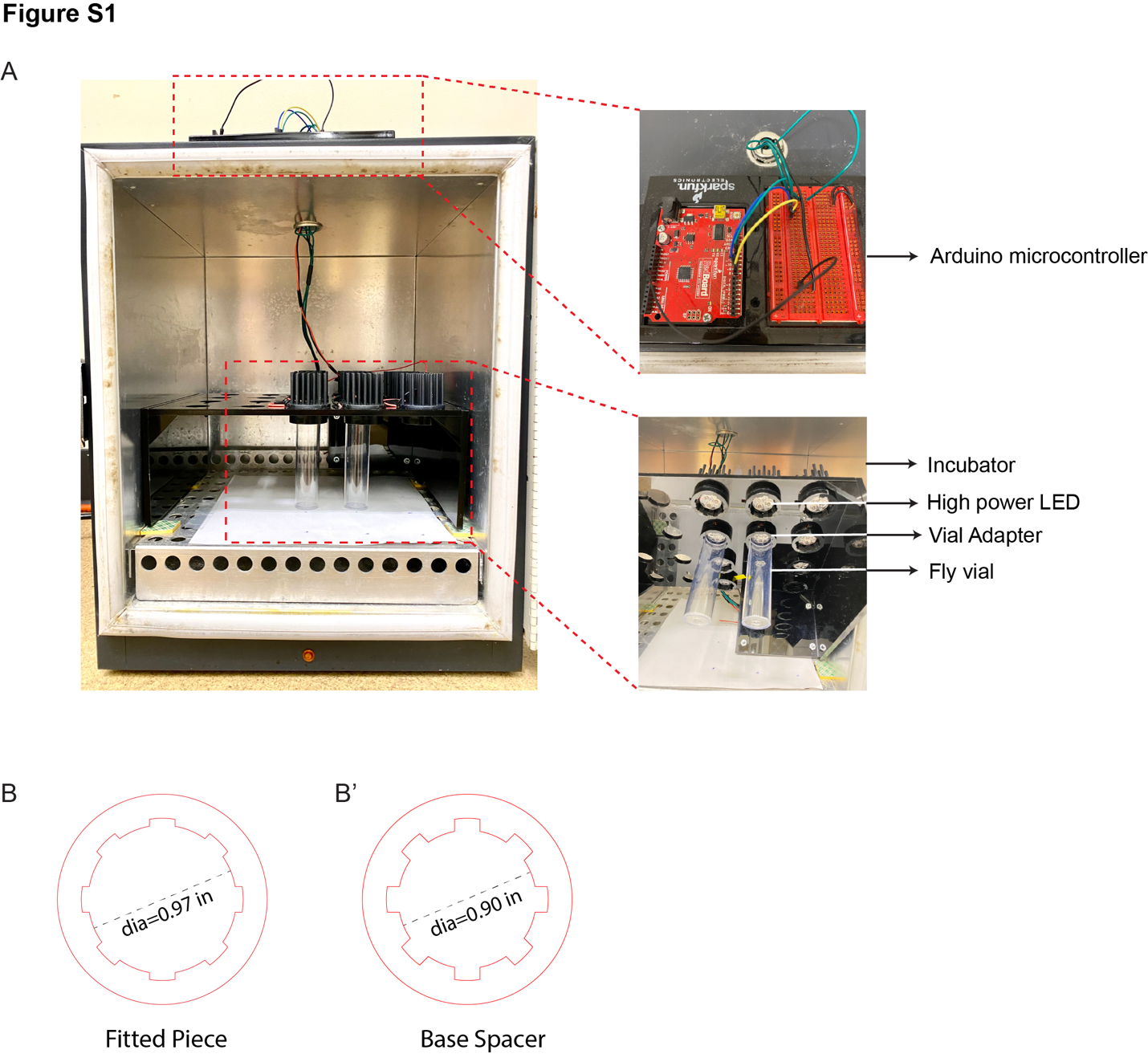
**

**Figure S1: The OMC setup and Scaled diagrams for the adapter ring setup**

(A) Photograph showing the main parts of the OMC setup, which include the incubator, Arduino board, fly vial adapter, and the high-power LEDs within the vial adapter. Refer to Figure 1A for more details.

(B-B’) The two rings were stacked such that the cogs of the Fitted piece (B) overlap the troughs in the Base spacer (B’) and then joined using acrylic cement to generate the holder. The inner diameter of the rings is 0.97 inches (for B) and 0.90 inches (for B’). Note: The stroke width of the rings has been increased from 0.001 to 0.25 for a better visibility in the manuscript figure. For laser cutting, the lines were kept at a dimension of 0.001 inches.

**
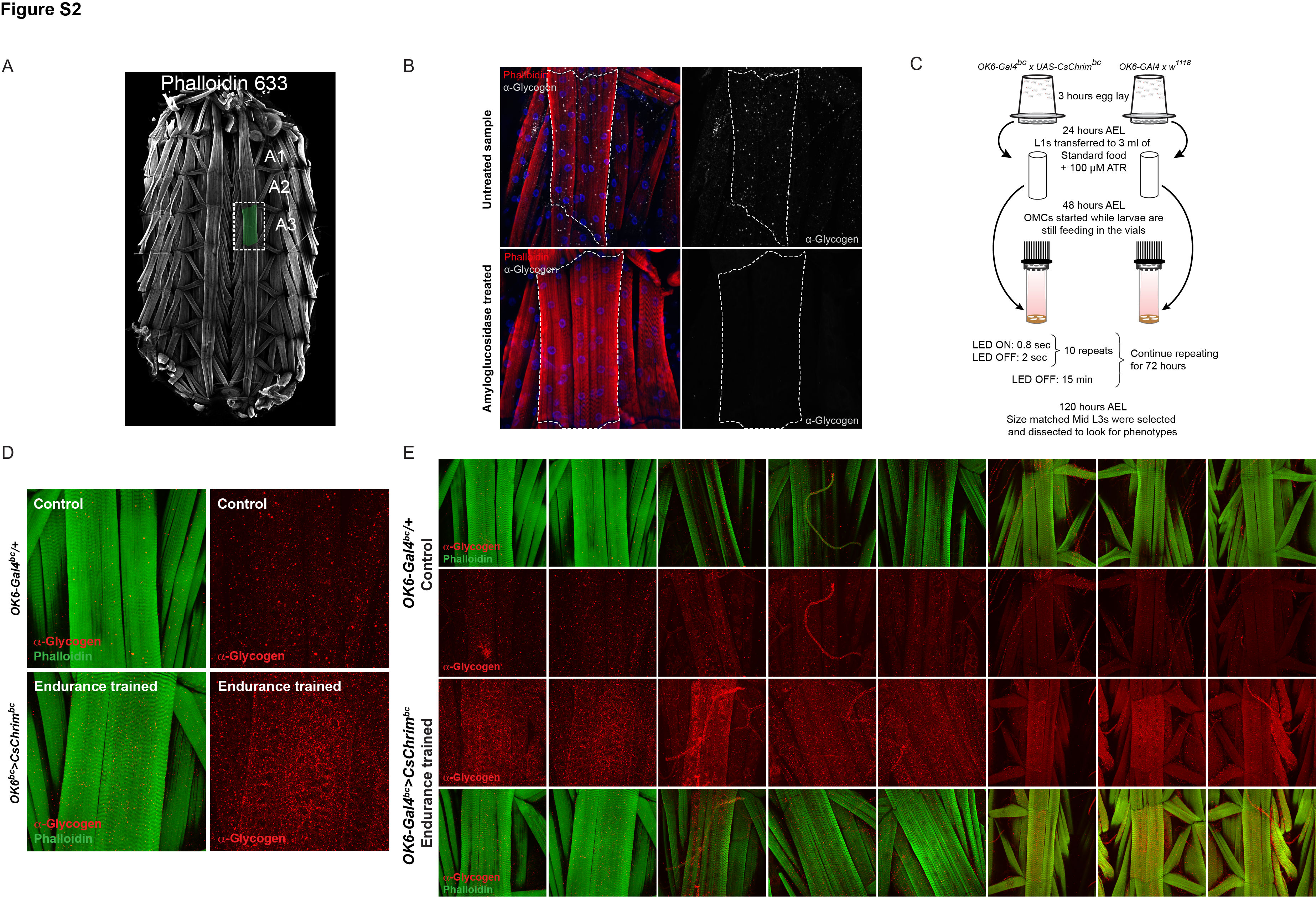
**

**Figure S2: Establishment of an endurance training regimen using OMCs**

1. Representative larval body wall muscle showing the muscle segment A3, which is used for imaging. Body wall muscle is stained with Phalloidin 633 (far red).
2. *w^1118^* larval body wall muscles stained with anti-glycogen antibody (α-Glycogen in grey), phalloidin-594 (red), and DAPI (blue). Sample in the lower panel was treated with amyloglucosidase for 20 min before staining with α-Glycogen antibody.
3. A schematic showing the experimental set up and frequency of OMCs used for modeling of endurance exercise.
4. Anti-Glycogen (α-Glycogen) staining of larval body wall muscles from control animals and animals that were endurance trained. Endurance trained animals show much stronger staining indicating increased glycogen storage in endurance trained muscles.
5. Anti-glycogen (α-Glycogen) staining of control and endurance trained larval body wall muscles. These are biological replicates (n=8) pertaining to (D). Note that the images in the second column are represented in (D).


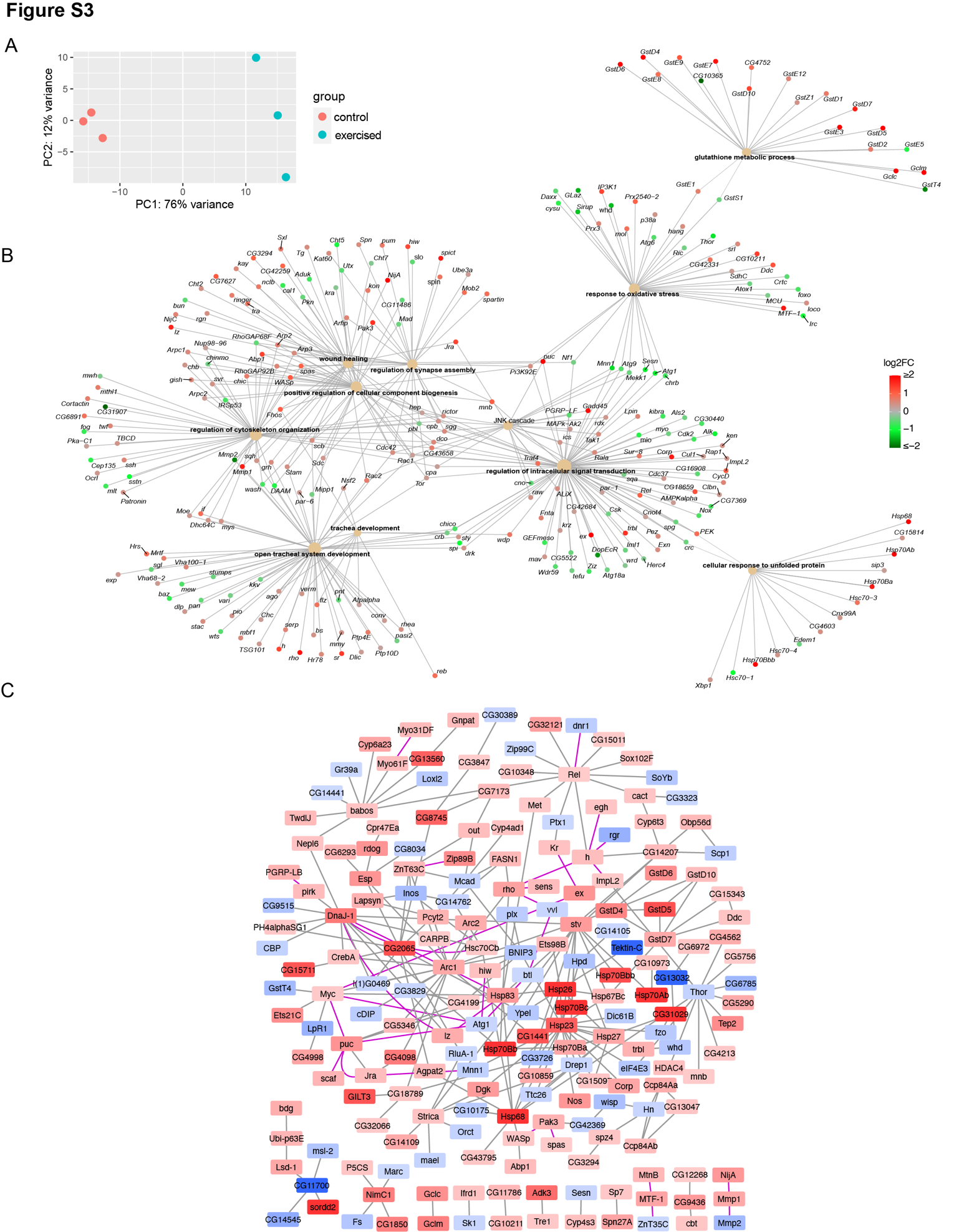


**Figure S3: Acute exercise affects genes involved in tracheal development, wound healing, oxidative stress, and glutathione metabolism**

1. Principal Component Analysis (PCA) showing the separation of control and exercised samples using top variance genes.
2. A cnet-plot showing the 10 selected significantly enriched GO terms with the relevant differentially expressing genes. The color of gene node represents the log2 value of fold change with the brightest red representing the log2-FoldChange ≥ 2 and the darkest green representing the log2FoldChange ≤-2
3. Gene interaction networks depicting the top differentially expressed (DE) genes with at least 2-fold change. The node color reflects the expression change (red for upregulated and blue for downregulated genes). The edge is either protein-protein interaction (PPI) or genetic interaction (GI) from MIST database (grey-PPI while pink- GI). The network is visualized using Cytoscape.

**Supplementary Table 1: Identification of Differentially Expressed Genes (DEGs) in acute exercise compared to controls.**

List of differentially expressed genes in exercised body wall muscle compared to controls.

**Supplementary Table 2: Identification of upregulated genes within the multiple GO terms related to morphogenesis.**

List of all upregulated genes of at least 1.5 fold change within the multiple GO terms that are related to morphogenesis.


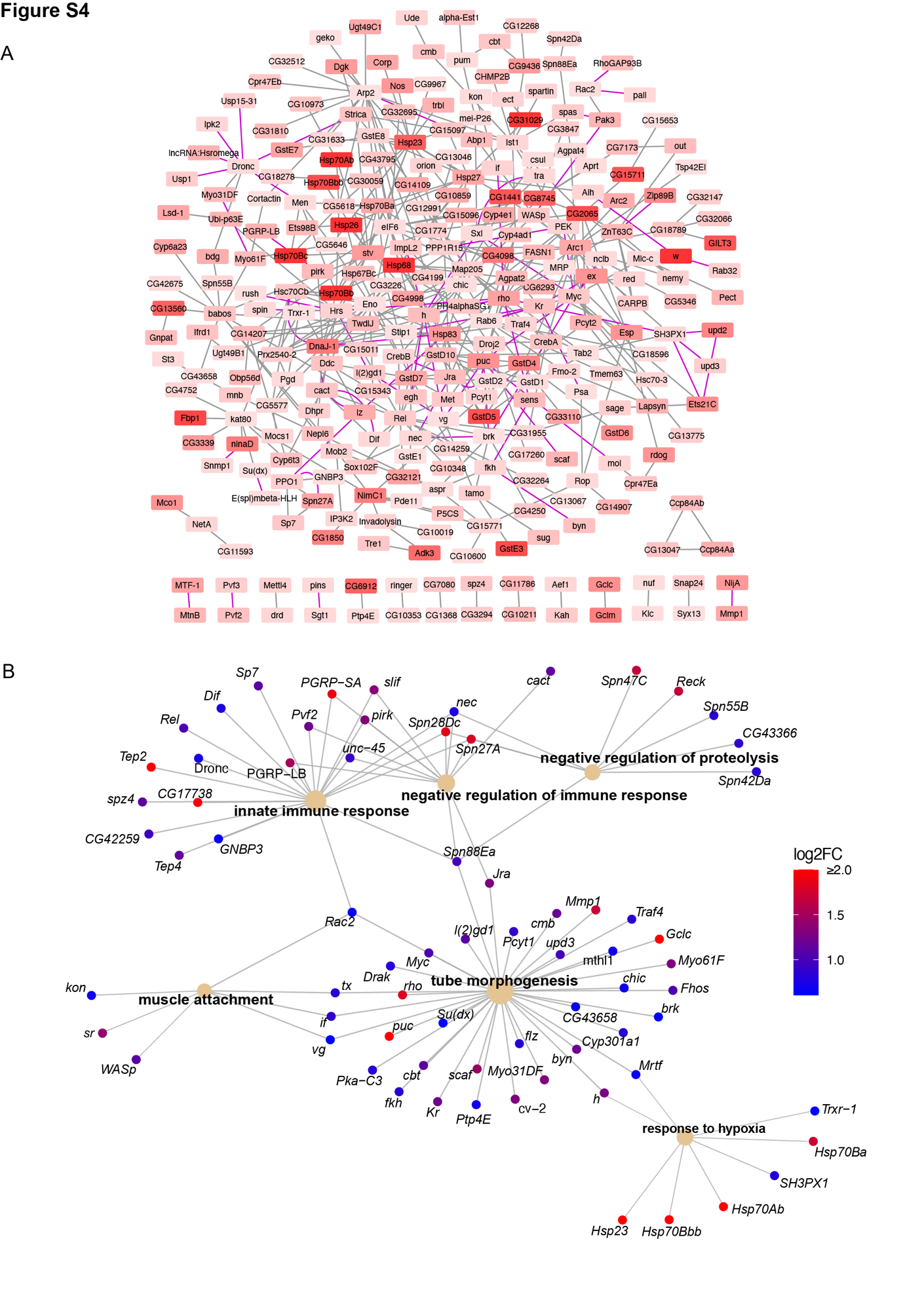


**Figure S4. Acute exercise upregulates genes involved in response to hypoxia, tube morphogenesis, and muscle attachment**

1. Gene interaction networks depicting the upregulated genes with at least 1.5-fold change. The node color in red reflects the expression change. The edge is either protein-protein interaction (PPI) or genetic interaction (GI) from MIST database (grey-PPI while pink- GI). The network is visualized using Cytoscape.
2. A cnet-plot showing additional 6 selected GO terms that were significantly enriched for genes that show an upregulation of 1.5 fold or more. The color of gene node represents the log2 value of fold change with the brightest red representing the log2-FoldChange ≥ 2 and the darkest blue representing a log2-FoldChange of 0.60.

**
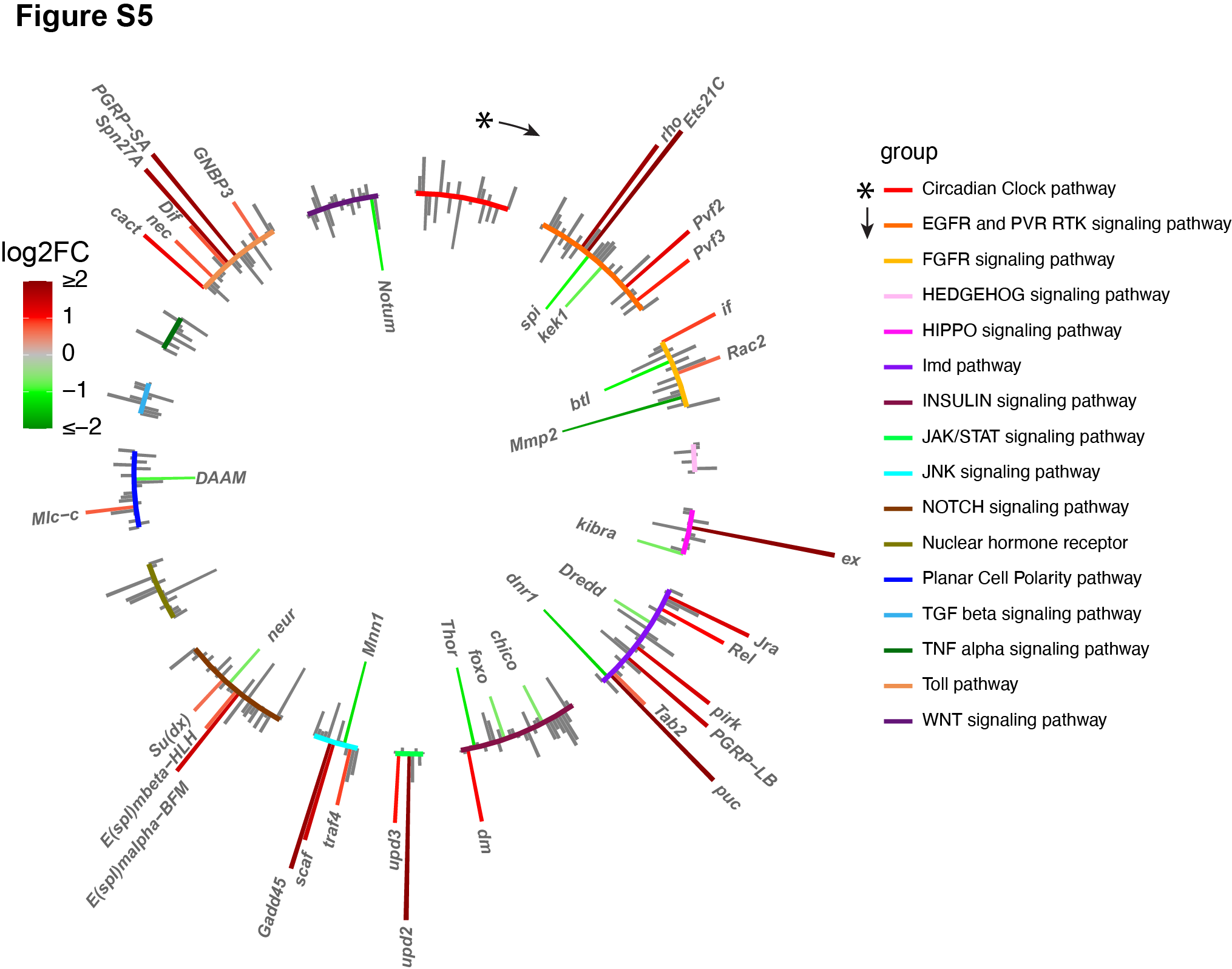
**

**S5: Changes in expression of core signaling pathway genes in response to acute exercise**

Genes that show a significant and more than 1.5 fold change in response to exercise are color coded with red showing an increase in expression and green a decrease in expression. Note that some genes are involved in more than one pathway, however, for simplicity of representation these genes have been shown for only one pathway.
